# A Summary of My Experience with Cataphoresis

**Published:** 1897-02

**Authors:** L. L Barber

**Affiliations:** Toledo, O.


### A Summary of my Experience with Cataphoresis.

BY L. L. BARBER, D.D S., TOLEDO, O.

Read before the Ohio State Society, December, 1896.

About six months ago I commenced to keep a record of cases operated upon with the different cataphoric apparatuses with a view, if possible, of establishing some guide to its future utility in dentistry.

1st. What cases are most favorable to its use?

2nd. What kind of medicaments were most efficient by its use?

3rd. What conditions were especially unfavorable to its use, etc.?

My record covers a little less than one hundred and fifty cases, and they have been so varied that without a correct record as to age, class of teeth, size of cavities, where located, susceptibility of patient to electricity, the noting of fear from pain, when able to do so, amount of time consumed for every case operated upon as compared to the approximated time it would have consumed without it, etc. I do not see how one could hope to arrive at anything like a true deduction as to whether cataphoresis is a benefit to them in the broad sense of the term or not. To me the keeping of a record has proven very instructive as well as interesting, and may prove so to some of those who are contemplating the use of some of the many cataphoric appliances upon the market.

It is probably a fact that any of the apparatuses made for the purpose will transfer medicaments to deep-seated tissues. There is a difference, however, in the amount of pain produced by some over others, even with the same amount of current and at the same pressure. It is very necessary that the operator have perect control over the current. Just how this is to be done I will let the electricians settle. However, I have found the current with some apparatuses very much more under control than with others.

Some patients are so sensitive to electricity that it is almost impossible to get enough current to carry the anæsthetic into the dentine without producing more pain than the patient cares to stand even with the most sensitively-controlled instrument. I have found some such cases for which I could find no other reason than these persons' natural susceptibility to the electrical current. On the other hand, many cases will be found which, to the beginner, might appear to be in the same class, but which, when better understood, can be attributed to various other causes, among which my record of cases show the following:

First, persons who are frightened. I have quite a number of cases on record where one, two, and in one case, as many as three attempts were made, at different sittings, before the patient could be brought to an understanding of the difference between the cold and slight pricking sensation attending the application of an electrical current, and pain. One case, upon which the third unsuccessful trial was made, now will not have a tooth filled without it. I have attached a report of this case. This case was a boy of eleven years. I think he did not experience anything but fear the first three times. One of the other very bad cases was not fear, for I found the person could feel my pulse, so to speak, so susceptible was she to electricity. I fastened the annode to the clamp upon the tooth, and that trouble was over. Another trouble I had when using the 110-volt current was, every now and again I thought the patient was going out of the fifth-story window. Finally I discovered that just before he was about to take his departure he would take hold of the iron bracket, arm or some metal part of the chair and so form a ground connection. That was something I was pleased to be able to avoid in the future. Even with a dry-cell battery you may find this same trouble, being due, as I believe, to some imperfection in some one or more of the cells. It is better to have each cell tried separately before connecting up, and thus be on the safe side.

Deductions show that very hard teeth are more tedious to control, and very much more time is required to produce the desired results owing to the fact of there being less animal matter, therefore, more resistance, and unless you are very careful you will put on pressure, or rather voltage, enough to produce pain by heat. Therefore keep the cavity wet (not the whole tooth), and do not try to get good results in a case of this kind as quickly as in a soft tooth.

Large labial cavities, according to the records kept, show, possibly, a larger percent of satisfaction than any other class. Those large, extremely-painful, and exceedingly-difficult labial cavities in the incisors and cuspids have been, in my hands, very satisfactory. However, in almost all classes of cases where the cavity was accessible and the condition of the pulp nearly normal, the work has been quite satisfactory.

The anæsthetic solutions that have been used by the writer include: hydrochlorate of cocaine in various strengths, from 4 percent up to 30, 50 and even 75 percent. Guaiacol, which is one of the phenols, is not as much of an escharotic as is creosote, of which it is an extract. But experiments tend to show that guaiacol has a coagulating action, and, therefore, does not permit of all the anæsthetic properties of the cocaine being brought out. By the use of most of the solutions of cocaine I have failed to get the phenomenal results that some have claimed.

Eucaine was used in quite a number of cases. I do not think, however, that better results were reached, except the anæsthesia produced proved to be more prolonged, if that could be claimed an advantage.

The solution with which the most satisfactory results have been obtained, according to the record kept in my office, is a 30-percent alcoholic solution of cocaine, or rather cocaine is soluble in alcohol one to three and one-half parts.

In looking over the records I find cases covering youth, manhood and old age, male and female, persons who were very susceptible to electricity and those who were *vice versa*, persons who were scared all but to death and those who were not frightened at all, persons upon whom it worked charmingly and those upon whom it did not work at all, except in a way one did not desire to have it. But the alcoholic solution of which I have spoken has the best record.

The cases recorded substantiate the therapeutic axiom that the degree of reaction is proportional to the degree of vitality, the greater the vitality the greater the reaction. Hence, any departure from the normal would retard the action of the cocaine solution used. So that, in a general way, it may be said that any condition of the pulp that lowers the physiological tone of it, will retard, not only the absorption of the cocaine, but also make the tubule fibrils and pulp harder to anæsthetize. Cocaine is a nerve-paralyzant when brought in contact with it, and then only. So, when used in a tooth without a nerve-exposure, we can hope for the anæsthetic effect only by the current carrying it through the tubuli. So, the ideal result will be less in proportion to the amount of lowering of the physiological tone of the fibrils.

My records show that dentine is anæsthetized primarily over the radius covered by the positive pole, because the current passes in the course of least resistance toward the apex of the root, through the tubuli and pulp canal. Thus, the larger surface being covered by the positive pole, the greater will be the surface obtunded. It is only secondarily that all of the dentine can be obtunded, but one can, by using a disk of metal over the saturated cotton, and then placing the platinum point upon the disk, get a larger current-covered surface, and thus secure better results. I hope and believe that there will soon be a better preparation for use in connection with the electrical current.

Messrs. Parke, Davis & Co. are now experimenting, and hope soon to give us something better than we have for this purpose.

We may expect the effect of drugs introduced into the dentine to be more lasting, owing to the absence of active fluid circulation there than in soft tissues.

Since writing the above I have had two cases I want to mention: One a boy twelve years of age—distal cavity in right superior central incisor and mesial approximate superior right lateral incisor. Both were extremely sensitive; by fitting a piece of platinum wire extending into each cavity and ten minutes' application of a current reaching 20 volts, I prepared both cavities with perfect comfort to the patient, and, mind, this was not a case of "cure worse than the disease," for there was (according to the boy), not the least pain during the process of obtunding. The other case was a man forty years of age, practiced law for some years, had nervous prostration, left his profession but has never fully regained control of his trouble. Most of the teeth he has lost has been on account of their extreme sensitiveness and his inability to stand the nervous shock, not on account of any thing else. On November 25th I operated upon the right second lower molar, it having a posterior prox. imal cavity, very much under the gum but not extending to the occluding surface. It also had a shallow but large buccal cavity mostly under the gum. It was impossible for me to prepare those cavities, so sensitive were they, and I could not get at them them to apply cocaine with the current, so I drilled a small hole into the occluding surface, and applied cocaine and current, and in twenty-one minutes was able to clean both cavities painlessly. The gentleman said it was entirely satisfactory during the application and excavation.

